# Development of a blended MOOC implementation model for English language learning: An interpretive structural modeling approach

**DOI:** 10.1371/journal.pone.0338980

**Published:** 2026-07-01

**Authors:** Anh Tuan Pham, Muhammad Ridhuan Tony Lim Abdullah

**Affiliations:** 1 Department of Management, Universiti Teknologi PETRONAS, Perak, Malaysia; 2 English Department, FPT University, Can Tho City, Vietnam; Middle Tennessee State University Jennings A Jones College of Business, UNITED STATES OF AMERICA

## Abstract

The integration of Massive Open Online Courses (MOOCs) into traditional English language education has created new opportunities for scalable, flexible, and inclusive learning. However, existing studies on MOOCs and blended learning remain largely descriptive and lack a structured framework that prioritizes key implementation elements for effective integration into formal education. This study develops a structured implementation model for blended MOOCs (bMOOCs) tailored to undergraduate English as a Foreign Language (EFL) instruction. This gap is particularly evident in specific contexts such as Vietnam, where institutional conditions, technological readiness, and learner characteristics significantly influence implementation effectiveness. Using the Interpretive Structural Modelling (ISM), the research identifies and validates 20 key sub-indicators grouped into three interconnected phases: planning, implementation, and evaluation. The model emphasizes the importance of analyzing learners’ backgrounds and motivations, setting clear learning objectives, and selecting appropriate materials and technologies. During implementation, the focus shifts to effective instructional strategies, learner participation, and technical support. The evaluation phase prioritizes formative assessment and timely feedback to promote continuous improvement and engagement. This learner-centered approach addresses common challenges in blended learning environments. The model offers practical guidance for stakeholders in EFL education, such as administrators, educators, and curriculum developers, seeking to enhance English language education through blended MOOCs. Practical implications for the implementation of blended MOOCs in EFL instruction are also addressed to enhance the quality of English language instruction in the digital age.

## 1. Introduction

Online courses have been widely implemented in educational settings due to advancements in digital technologies [1]. This trend is gaining popularity in developed countries but remains limited in developing contexts due to costs and educational inequalities [2]. High-quality online programmes are often delivered by prestigious institutions such as Harvard, Stanford, Cambridge, and Oxford, which employ effective pedagogical and strategic approaches to enhance educational quality and sustainability [3,4].

Therefore, developing countries are encouraged to adopt online learning modes to improve educational quality [2]. Massive Open Online Courses (MOOCs), introduced by Siemens and Downes in 2008, have played a key role in the transformation of online learning [5]. During the COVID-19 pandemic, MOOCs have become an important alternative to face-to-face instruction and supported the continuation of education during institutional closures [6]. In the post-pandemic period, many institutions have adopted blended learning models that combine online and classroom-based activities [7–9]. However, concerns about limited interaction and feedback in fully online MOOCs have led to the emergence of blended MOOCs (bMOOCs), which integrate MOOC content into face-to-face classrooms to address these limitations [10,11].

Previous studies report that blended MOOCs can enhance learner–instructor interaction, learner autonomy, and academic performance, indicating their potential to improve engagement and learning outcomes in higher education [12,13,14–17]. In EFL education, MOOCs have been widely used to extend language practice beyond the classroom and are considered a useful tool for English language programmes [10,18–21]. Research further indicates that MOOC-based blended learning can improve learner engagement, motivation, and course achievement in EFL contexts [22–25]. However, despite these benefits, existing studies primarily focus on the effectiveness of MOOCs or blended learning in isolation, with limited attention to how key implementation elements should be systematically structured and prioritized. This lack of a structured implementation framework makes it difficult for institutions to effectively integrate MOOCs into formal teaching practices.

In Vietnam, English is a compulsory subject and an essential skill for academic and professional development [26,27]. However, traditional classrooms face challenges such as large class sizes and limited practice time, which restrict opportunities for meaningful language use [28–32]. Although MOOCs provide flexible access to English learning resources, they often lack sufficient interaction and feedback [33–36]. Additionally, institutional constraints, technological readiness, and learner characteristics in the Vietnamese context further complicate the effective integration of MOOCs into formal curricula. Blended MOOCs, therefore, present a promising solution for improving learner engagement and language development in EFL education [22,24,25]. Despite growing interest in this approach, there is still a lack of a comprehensive and hierarchical framework that identifies the interrelationships among critical implementation factors, particularly within specific educational contexts such as Vietnam. This study aims to address this gap by proposing a structured blended MOOC implementation model for undergraduate EFL education. Unlike previous studies that primarily focus on individual aspects of MOOCs or blended learning, this study develops a comprehensive and hierarchical implementation model using the ISM and MICMAC approaches. The study uniquely identifies the interrelationships among indicators and classifies them based on driving and dependence power, providing a structured framework for decision-making in EFL blended MOOC contexts.

## 2. Literature review

### 2.1. Blended Learning

Blended learning (BL) refers to an instructional approach that combines in-person classroom experiences with online learning components within a single course [37]. Educational institutions have increasingly adopted this model due to its capacity to enhance student engagement, interaction, and persistence when compared to conventional online learning formats [38–41]. One of the major strengths of BL is its adaptability, offering benefits to both learners and instructors since it promotes student autonomy, improves academic performance, and helps create a more integrated learning mode between traditional and virtual teaching methods [42].

Additionally, BL enables learners to access a wide range of learning materials at any time and from anywhere, making it easier for them to study at their own pace [43]. For instructors, this mode may help reduce teaching load and create opportunities for more efficient classroom management [44]. Regarding advantages, BL is considered an effective teaching method in higher education contexts [45]. However, there remains a lack of empirical research into how BL is implemented across different academic programs and course types, particularly from the perspective of institutional leaders.

There is also ongoing debate regarding the balance between online and face-to-face instruction. Some suggest a ratio of 30% in-person learning to 70% online [46], while others propose a 20/80 format [44]. These differences highlight the need for more comprehensive guidelines on BL implementation in higher education.

### 2.2. Blended MOOCs

To improve the quality of university education, blended MOOCs (bMOOCs) have emerged as a promising mode that integrates MOOC instruction with classroom-based one [36]. In a bMOOC setting, educators either design their own digital materials or utilize existing MOOC resources created by experts. This model enhances interaction, supports student-centered learning, facilitates better assessment and feedback, and accounts for diverse learning styles and behavior patterns [36]. Moreover, by shifting some content delivery online, instructors can focus classroom time on discussions and problem-solving activities [47]. However, learners often find this model unfamiliar and are required to take more responsibility for their learning and class engagement [48]. Despite its potential, research on how blended MOOCs can be effectively applied in specific programs such as EFL remains limited.

### 2.3. Blended MOOCs in English language learning

In the context of English language education, the use of blended MOOCs in EFL classrooms is still underexplored. English, which is a skill-based subject, presents challenges when integrating MOOCs with conventional classrooms [49]. Besides, students with limited English proficiency often struggle to follow online lectures or engage in peer communication, which can result in frustration and a preference for traditional classroom settings [50]. While MOOCs are generally suitable for teaching receptive skills such as listening and reading, they are less effective for productive skills such as writing and speaking, which require more direct teacher involvement and feedback [51,52]. Despite these challenges, blended MOOCs retain their effectiveness in English teaching and learning [22,23,53]. Therefore, more investigation is needed to develop a context-specific implementation framework that addresses these gaps.

### 2.4. Indicators and sub-indicators for blended MOOC implementation in EFL programs

**Fig 1:**
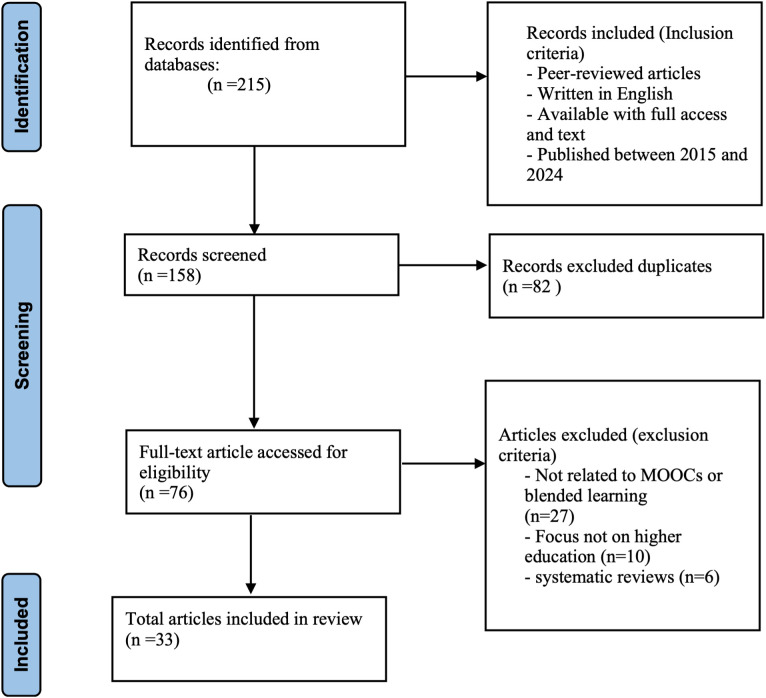
A flow diagram of the study selection based on PRISMA.

A comprehensive literature review was conducted to explore the primary indicators for a blended MOOC implementation framework for English courses. To review the indicators for blended MOOC implementation for EFL programmes, the Preferred Reporting Items for Systematic Reviews and Meta-Analyses approach (PRISMA) was employed as guidelines for the selection of indicators, which follows four phases: identification, screening, eligibility, and analysis [54]. The sources included journal articles, conference proceedings, extracted from Google Scholar, Elsevier, and Web of Science, with a publication range from 2014 to 2024. The same string of search words: “MOOCs or Massive Open Online Courses” AND “blended learning or blended classes” AND “EFL or English as a Foreign Language” AND “indicators or sub-indicators or success factors” was used for each of the databases. In Fig 1, this PRISMA flow diagram illustrates the systematic process of selecting articles for a review. Initially, 215 records were identified from databases based on inclusion criteria such as peer-reviewed status, English language, full-text availability, and publication between 2015 and 2024. After screening, 82 duplicate records were excluded, leaving 158 records for further assessment. From these, 76 full-text articles were accessed to determine eligibility. Articles were then excluded if they were not related to MOOCs or blended learning (27 articles), did not focus on higher education (10 articles), or were systematic reviews (6 articles). Finally, 33 articles met all criteria and were included in the final review.

The literature was analysed and synthesized by themes and was then categorized into six key indicators: analysis of learners, objectives, course materials and technology, teaching methodologies, learner participation, and feedback and assessment, with 21 sub-indicators in total.

As blended learning continues to shape the future of English language education, the integration of MOOCs (Massive Open Online Courses) into undergraduate English programs demands a structured and context-sensitive implementation model. Drawing on existing research, this review synthesizes six key indicators and their sub-indicators that underpin the successful integration of blended MOOCs in EFL learning environments: analysis of learners, objectives, course materials and technology, teaching methodologies, learners’ participation, and feedback and assessment.

The first indicator is the “analysis of learners”, which involves understanding students’ backgrounds, motivations, and digital familiarity with MOOCs. Clearly defined learner backgrounds, including age, learning styles, and English proficiency, are essential to tailoring instruction to diverse needs [55–58]. In MOOC settings, students’ familiarity with digital platforms also plays a critical role, as low digital literacy can hinder engagement and progress [59,60]. Moreover, intrinsic motivation and goal-setting are strongly associated with learner persistence in online environments [23,33,58], especially in self-directed MOOC models where autonomous learning is essential.

The second indicator focuses on learning “objectives”, which must be measurable and aligned with student proficiency levels. Clear objectives provide a guide for both learners and instructors, directing the selection of materials and assessments [10,61]. In blended MOOC environments, it is also crucial that learning outcomes are achievable within a defined timeframe [62,63] and aligned with learner needs and abilities [64], allowing for realistic goal-setting and meaningful progress.

“Course materials and technology” is the third indicator, which addresses both access and pedagogical relevance. The availability of MOOCs through learning management systems (LMS), social platforms, and open resources enhances flexibility and accessibility [10,65]. However, materials must also be linguistically appropriate and engaging. Studies emphasize the importance of using various teaching content, such as videos, quizzes, and readings, which support different English language skills and learner preferences [23,33].

“Teaching methodologies” then highlights the importance of balancing online self-paced activities with offline mentoring [36,66]. Blended instruction in EFL contexts must also draw from effective language teaching methods [22,67] and incorporate multimedia and interactive tools to support comprehension [68]. Learning support mechanisms, such as technical or academic support, are essential for maintaining learner engagement in digital environments with MOOCs [69,70].

“Learners’ participation” is critical for engagement and interactions. Regular interaction with peers and instructors enhances motivation and learning outcomes [67,71]. Offline mentoring sessions [72], collaborative learning activities [12], and self-regulation skills [73,74] are all important indicators of active participation in blended models.

Lastly, the indicator “feedback and assessment” plays a central role in monitoring progress and evaluation. Effective MOOC-based EFL instruction includes both formative and summative assessment strategies [71,75] and offers feedback through peer, instructor, or automated systems [67]. Instant feedback can inform syllabus revisions [76], while offline final exams may be used to assess learning outcomes [77,78], particularly in formal university settings Table 1. In general, these six indicators provide a comprehensive framework for designing and evaluating blended MOOC implementation in undergraduate English learning.

**Table 1:** The proposed indicators and sub-indicators for a blended MOOC implementation model for English Learning.

Indicators	Sub-indicators	Representative literature
Analysis of learners	“Clearly defined learners’ backgrounds (age, learning styles, and current level of English proficiency)”	[55], [56–58]
	“Learners’ familiarity with MOOC platforms”	[59], [60]
	“Learners’ motivation and goals to learn English”	[23], [33,58]
Objectives	“Clearly defined and measurable learning objectives”	[10], [61]
	“Achievable learning outcomes within a time frame”	[62], [63]
	“The alignment of learning outcomes with students’ needs and levels”	[62], [64]
Course materials and technology	“Availability and accessibility to MOOCs (e.g., LMS, online materials, social blogs, etc.)”	[10], [65]
	“The suitability of teaching materials (textbooks, videos, quizzes, etc.)”	[10], [23]
	“Variety of teaching content that develops English language skills”	[23], [79]
Teaching methodologies	“Suitable proportion of online (self-paced) activities and offline mentoring activities”	[23], [80]
	“Suitability of English language teaching methodologies”	[22], [36,67]
	“Effectiveness of using teaching aids (visual aids, multimedia, instructional materials)”	[36], [68]
	“Availability of effective learning support (technical issues, assignment issues, etc.)”	[67], [69,70]
Learners’ participation	“Level of interaction between students and other peers, instructors”	[67], [71,81]
	“Learners’ participation in offline mentoring sessions”	[72], [82]
	“Effective collaboration in interactive activities”	[12], [67]
	“Effective self-regulated learning in online and offline English classrooms”	[58], [73,74]
Feedback and assessment	“Appropriate assessment methods (formative and summative assessment)”	[63], [71,75]
	“Instructor, peer feedback and online auto-feedback on student progress in specific English skills”	[67]
	“The revision of course syllabi based on feedback and performance data”	[76]
	“The offline final examinations to re-evaluate students’ English performance”	[72], [77,78]

## 3. Methodology

The development of a blended MOOC implementation model is based on the integrated view and experts’ decisions, so interpretive structural modeling (ISM) was employed in this study to develop the model. Accompanied by ISM, the Fuzzy Delphi method was initially applied to assess the indicators and sub-indicators for blended MOOC implementation. ISM is used to examine the relationships among blended MOOC indicators and sub-indicators as well as a structural model based on the relationships for blended MOOC implementation for an undergraduate EFL programme. ISM was developed by Warfield to analyze and solve complex issues in socio-economics [83]. However, past studies also proved that ISM was widely used in the fields of knowledge management, engineering, and information systems [84–87]. In higher education, ISM was also adopted to tackle the issues related to technical and social education [88,89]. ISM is useful to help the participants in groups structure their views and others to reach a consensus through a mathematical language application, such as words, diagrams, and mathematics [90]. Therefore, in this study, the employment of ISM to develop a blended MOOC implementation model for undergraduate EFL learning is applicable.

In the decision-making process, the Fuzzy Delphi method was employed. 15 selected experts were involved to give opinions on the importance of the indicators and sub-indicators through online questionnaires. In this study, one round of the FDM was conducted, as consensus among the experts was achieved in the first round. The level of agreement was determined based on predefined threshold criteria, including the threshold value (d ≤ 0.2) and the agreement level (A ≥ 0.5). Since all indicators satisfied these conditions, no additional rounds were required.

Ethical approval for the study was granted by the Research Ethics Committee of FPT University under reference number 20250205.02. Written informed consent was obtained from all participants via a self-administered online questionnaire. Participants were assured that their responses would be used solely for research purposes, and all data were handled with strict confidentiality throughout the study. The qualified experts must have experience in the related fields of MOOC and blended learning. The selection of experts should follow the criteria [91]: 1) The experts should have good knowledge of the topic being studied, 2) They should have experience working in the field, with at least five years of related experience, 3) The experts need to be fully committed and take part in the study until it is completed, and 4) There should be no personal connection or interest that could affect their opinions or cause bias in the study. The experts included diverse stakeholders, such as administrators, lecturers, curriculum developers, ICT engineers, MOOC instructors, and EFL undergraduates. Although experts should have rich experience in working with MOOCs, the undergraduate students were considered experts as their voices and experience were valuable, and they are the main users of MOOC-based blended learning, so their perspectives provide practical knowledge about learning experiences that may not be fully captured by administrators or lecturers alone [92]. In this study, undergraduate participants were therefore treated as experiential contributors whose views reflected real learner interaction with MOOCs, platform usability, motivation, and assessment practices.

The experts’ demographic information is displayed in Table 2.

**Table 2:** Demographics of selected experts.

Expert	Position	Qualification	Experience
Expert 1	Director of English language program	Ph.D	~ 25 years
Expert 2	Head of MOOC curriculum developer	Ph.D	~ 20 years
Expert 3	English lecturer	Ph.D	~ 18 years
Expert 4	English lecturer	Master of TESOL	~ 10 years
Expert 5	English lecturer	Master of TESOL	~ 10 years
Expert 6	English curriculum and program developer	Master of Arts	~ 8 years
Expert 7	English curriculum and program developer	Master of Arts	~ 10 years
Expert 8	ICT engineer in English education program	Master of Science	~ 11 years
Expert 9	ICT engineer in English education program	Master of Science	~ 7 years
Expert 10	English MOOC instructor	Master of TESOL	~ 11 years
Expert 11	English MOOC instructor	Master of TESOL	~ 16 years
Expert 12	English MOOC instructor	Master of TESOL	~ 21 years
Expert 13	English language student	Undergraduate	2nd year
Expert 14	English language student	Undergraduate	2nd year
Expert 15	English language student	Undergraduate	4^th^ year

**Fig 2:**
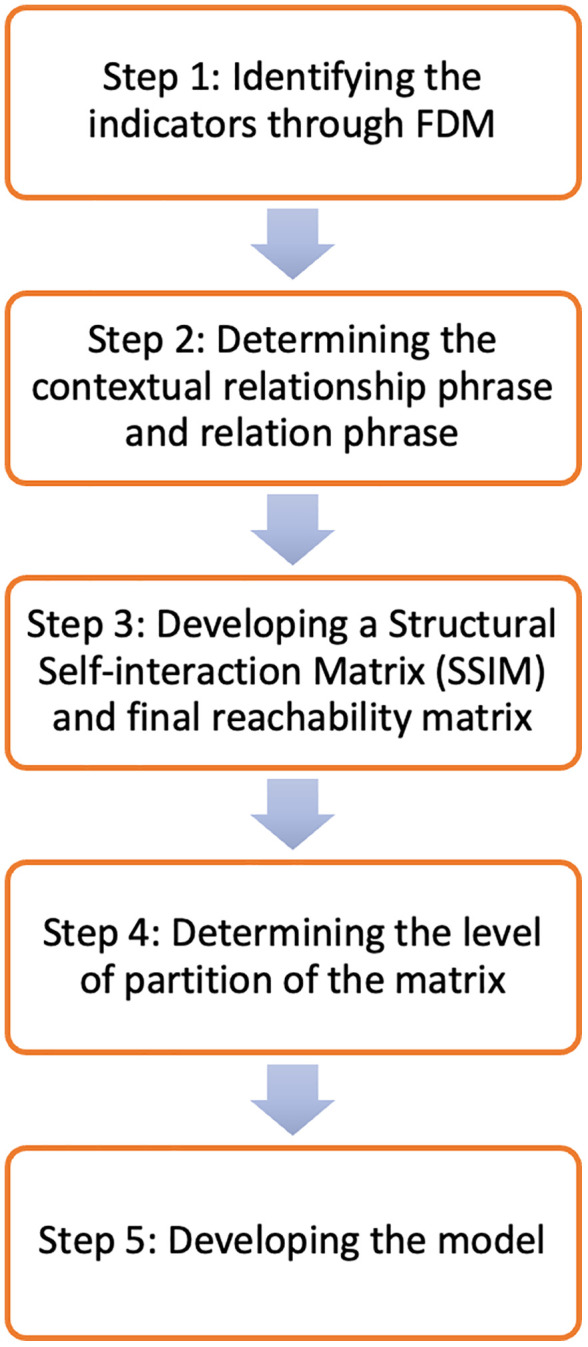
Data collection procedures.

Data collection procedures were carried out at the studied institution from August 10, 2024 to May 25, 2025, including surveys and interviews. The data were analyzed based on 5 steps, as in Fig 2.

*Step 1: Identifying the indicators through Fuzzy Delphi method (FDM)*

To assess the indicators for a blended MOOC implementation model for English language learning, the Fuzzy Delphi Method (FDM) was chosen due to its effectiveness in minimizing subjectivity and ambiguity while promoting agreement among experts. This method is frequently employed to identify relevant ideas or variables linked to a particular issue or challenge. Originally introduced by Kaufmann and Gupta, FDM is a well-regarded tool in decision-making processes [93]. It combines elements from fuzzy set theory and the traditional Delphi method [94]. Essentially, FDM merges the structured, iterative process of the Delphi technique with the flexibility of fuzzy logic to support more accurate expert judgment. The FDM itself relies on multiple rounds of surveys to gather and refine expert opinions on a specific topic [95]. By integrating fuzzy logic, FDM enables experts to express their views in a more nuanced way, beyond simple yes/no answers, making it especially useful for exploring the complex needs involved in implementing blended MOOCs for English language education. The FDM involves several steps: from identifying indicators taken from the literature to assessing these indicators based on fuzzy logic. The defuzzification value (A) was used to rank the indicators, following the formula: A = 1/3*(m1 + m2 + m3). The assessment of the indicators and sub-indicators was based on a 7-point Likert scale questionnaire (between 1 = extremely not important and 7 = extremely important). The linguistic scale was employed to compute the data (see Table 3). To examine whether an indicator is accepted, the threshold value of consensus is achieved through d and is used to measure agreement between the evaluations made by the experts. The consensus value should have a cut point of 0.2 or below to define group consensus. However, the total consensus must exceed 75% for validation purposes [96,97]. If this threshold is not met, additional rounds of FDM are required to achieve satisfactory agreement among the experts. Therefore, the 75% consensus level in obtaining the results ensures that the findings are reliable and valid.

**Table 3:** The linguistic scale for the fuzzy Delphi method questionnaire.

7-point linguistic scale	Fuzzy scale (m1, m2, m3)
1. extremely not important	0.00, 0.00, 0.10
2. not important	0.00, 0.10, 0.30
3. somewhat not important	0.10, 0.30, 0.50
4. neutral	0.30, 0.50, 0.70
5. somewhat important	0.50, 0.70, 0.90
6. important	0.70, 0.90, 1.00
7. extremely important	0.90, 1.00, 1.00

*Step 2: Determining the contextual relationship phrase and relation phrase*

The experts identified and agreed upon the contextual relationship phrase “In order to develop a blended MOOC implementation model for English learning, the indicator” and the phrase “MUST be conducted before” is the relation phrase. As an example, two indicators “i” and “j” are determined through the statement “In order to develop a blended MOOC implementation model for English learning, the indicator “i” MUST be conducted before the indicator “j”. Experts’ votes “Yes” or “No” were used to determine the relationships among the indicators.

*Step 3: Developing a Structural Self-interaction Matrix (SSIM) and final reachability matrix*

SSIM is used to represent the relationships and interactions among the elements (indicators) in a system. It is a square matrix that represents the self-interactions or feedback of the elements (indicators) in the system [96]. Specifically, the associated direction of the relationships between two elements (e.g., element i and j) is denoted by using four symbols: a) V for the relation from element i to j; b) A for the relation from element j to i; c X for both direction relations and d) O for no relation between i and j.

The construction of the reachability matrix was to classify the elements into different levels. This was achieved based on SSIM (Step 2) by replacing V, A, X, and O as 1 and 0, as given below. The replacement of 1s and 0s are as follows:

“If the entry (i, j) in SSIM is V, the entry (i, j) in the target matrix becomes 1, and the entry (i, j) becomes 0,

If the entry (i,j) in SSIM is A, the entry (i,j) in the target matrix becomes 0, and entry (i, j) becomes 1,

If the entry(i,j) in SSIM is X, the entry (i,j) in the target matrix becomes 1 and the entry (i, j) also becomes 1, and

If the entry (i, j) in SSIM is O, the entry (i, j) in the target matrix becomes 0, and the entry (j, i) also becomes 0”.

The ISM software helps to generate the model based on the concept of paring comparison and transitive logic. The transitive logic for any of three elements (a, b, and c) is drawn: a ◊ b (a has the relation to b); b ◊c (b has the relation to c); and a ◊ c or a◊ b ◊ c (a has the relation to c). The output of this step is also known as the conical matrix.

*Step 4: Determining the level of partition of the matrix*

Using the reachability matrix generated in Step 3, the elements were categorized based on their influence. This classification relied on two key sets for each element: the reachability set and the antecedent set. The reachability set includes the element itself and all other elements it can help to achieve. In contrast, the antecedent set consists of the element and others that contribute to its accomplishment. This categorization is crucial for constructing the model, as it assigns elements to hierarchical levels. The process involved identifying the intersection between the reachability and antecedent sets. Elements found in both sets were designated as occupying the upper levels within the Interpretive Structural Modelling (ISM) hierarchy, indicating their prominent role in influencing other factors.

*Step 5: Developing the model*

To categorize the indicators—a conical matrix (or finalized reachability matrix) was used in conjunction with the Matrice d’Impacts Croisés Multiplication Appliquée à un Classement (MICMAC) method. Originally introduced by Duperrin and Godet, MICMAC serves as a structured analytical tool designed to examine both direct and indirect relationships among variables and evaluate their levels of influence and interaction [98]. The central aim of MICMAC analysis is to explore how much influence a variable exerts (driving power) and how much it relies on others (dependence power) [99].

These two values—driving and dependence—are calculated from the finalized matrix by summing the values in each row (to determine influence) and each column (to assess dependence). Once calculated, the data can be plotted on a two-dimensional grid known as the driver-dependence diagram. In this diagram, the horizontal axis represents how dependent a variable is, while the vertical axis reflects how much influence it exerts on other elements.

Based on these two dimensions, elements are grouped into four main categories:

1) Autonomous cluster: These indicators have minimal influence and low dependence, and they tend to be weakly connected to the overall system.
2) Independent cluster: These indicators have a strong influence but low dependence, meaning they significantly drive the system and are often considered key or core activities.
3) Dependent cluster: These indicators rely heavily on others but exert little influence themselves.
4) Linkage cluster: These indicators show both high influence and high dependence, making them volatile and highly interconnected; changes to these elements can have wide-ranging effects, including feedback loops that affect the element itself.

## 4. Results

### 4.1. Identifying and assessing the indicators and sub-indicators for a blended MOOC implementation model for English language learning

As per the consensus from the panel of experts, 20 out of 21 elements were accepted as the key sub-indicators for a blended MOOC implementation framework. However, one sub-indicators “The revision of course syllabi based on feedback and performance data” was rejected by the experts (A = .611, d = .260, 60% of expert consensus).

Among the most critical sub-indicators identified, the expert consensus validated the importance of motivation in learning environments, so learners’ motivation was ranked as the most important. The element “learners’ motivation and goals to learn English” was most agreed upon (A = 0.893; d = 0.060, consensus = 100%) that motivated learners are more likely to involve themselves and thus gain better results in English blended MOOCs and also consistent with earlier investigations stating that intrinsic motivation is the basis for proper access because it leads to sustained use and higher completion rates in online environments.

The second most important sub-indicator (A = 0.860; d = 0.103) is “clearly defined learners’ backgrounds”, with a consensus of 80%, which includes understanding the learners’ ages, preferred methods of learning, and degrees of English ability. This element emphasizes how important it is to tailor course design to each learner’s specific needs.

“The suitability of teaching materials such as textbooks, videos, and quizzes” was ranked third by an A value of 0.856, a threshold “d” of.031, and 93% expert consensus. Therefore, resources of teaching materials are essential for keeping students interested by ensuring their quality as well as their compliance with pedagogic demands. “The variety of teaching content that develops English language skills” stood in fourth place (A = .842, d = .069, 93% expert consensus), following closely after the appropriateness of teaching materials. This emphasizes the need for integrating various teaching modalities to serve different learners’ needs and enhance their outputs.

Eighty percent of the experts agreed that the element “clearly defined and measurable learning objectives” was essential, ranking fifth (A = .833, d = .085). In the context of blended MOOCs, clearly defined objectives let learners achieve the goals of the course, how they will be assessed, and what they are expected to achieve. They also provide instructors with a parameter for assessing students’ achievements against the learning outcomes intended for the course Table 4.

**Table 4:** The consensus of sub-indicators for blended MOOC implementation for English learning.

No	Indicator and sub-indicators	Threshold value (d)	Expert agreement (%)	df value (A)	Ranking	Expert Consensus
1	“Clearly defined learners’ backgrounds (age, learning styles, and current level of English proficiency)”	0.103	80%	0.860	2	Accept
2	“Learners’ familiarity with MOOC platforms	0.183	80%	0.738	19	Accept
3	Learners’ motivation and goals to learn English”	0.060	100%	0.893	1	Accept
4	“Clearly defined and measurable learning objectives”	0.085	80%	0.840	5	Accept
5	“Achievable learning outcomes within a time frame”	0.081	80%	0.833	6	Accept
6	“The alignment of learning outcomes with students’ needs and levels”	0.138	93%	0.798	9	Accept
7	“Availability and accessibility to MOOCs (e.g., LMS, online materials, social blogs, etc.)”	0.145	93%	0.787	11	Accept
8	“The suitability of teaching materials (textbooks, videos, quizzes, etc.)”	0.031	93%	0.856	3	Accept
9	“Variety of teaching content that develops English language skills”	0.069	93%	0.842	4	Accept
10	“Appropriate proportion of online (self-paced) activities and offline mentoring activities”	0.190	80%	0.749	18	Accept
11	“Suitability of English language teaching methodologies”	0.147	93%	0.776	14	Accept
12	“Effectiveness of using teaching aids (visual aids, multimedia, instructional materials)”	0.125	100%	0.789	10	Accept
13	“Availability of effective learning support (technical issues, assignment issues, etc.)”	0.098	100%	0.822	7	Accept
14	“Level of interaction between students and other peers, instructors”	0.193	80%	0.760	17	Accept
15	“Learners’ participation in offline mentoring sessions”	0.147	93%	0.776	15	Accept
16	“Effective collaboration in interactive activities”	0.165	87%	0.784	12	Accept
17	“Effective self-regulated learning in online and offline English classrooms”	0.165	87%	0.784	13	Accept
18	“Appropriate assessment methods (formative and summative assessment)”	0.168	87%	0.762	16	Accept
19	“Instructor, peer feedback and online auto-feedback on student progress in specific English skills”	0.128	93%	0.809	8	Accept
20	“The revision of course syllabi based on feedback and performance data”	0.260	60%	0.611	21	Reject
21	“The offline final examinations to re-evaluate students’ English performance”	0.074	87%	0.731	20	Accept

At the bottom of the ranking is the sub-indicator of “the offline final examinations to re-evaluate students’ English performance.” This indicator was rated the lowest (A = 0.731) with a 87% consensus among the experts, thus making it the least prioritized aspect in the bMOOC framework. While offline final exams are usually viewed as the traditional mainstay of language learning assessments, their relevance in the blended MOOC context has increasingly been questioned.

To examine whether student participation affected the stability of the indicator structure, a brief subgroup sensitivity comparison was conducted between academic experts (n = 12) and student participants (n = 3). Both groups accepted the same 20 sub-indicators and rejected the same sub-indicator. The overall defuzzified mean values were highly similar (academic experts: A = 0.803; students: A = 0.794), with a negligible difference (ΔA = 0.009). The general ranking patterns remained consistent. This indicates that the indicator synthesis was stable and was not disproportionately influenced by student-only perspectives (See Table 5).

**Table 5:** Subgroup sensitivity comparison between academic experts and student participants.

Subgroup	N	Mean A	Accepted sub-indicators	Rejected Sub-indicators	Ranking pattern
Academic experts	12	0.803	20/21	1/21	consistent
Undergraduates	3	0.794	20/21	1/21	consistent

### 4.2. Determining the contextual relationship phrase and relation phrase

The experts identified and agreed upon the contextual relationship phrase “In order to develop a blended MOOC implementation model for English learning, the sub-indicator” and the phrase “MUST be conducted before” is the relation phrase. As an example, two sub-indicators “b” and “c” are determined through the statement “In order to develop a blended MOOC implementation model for English learning, the sub-indicator “a” MUST be conducted before the sub-indicator “b”. Experts’ votes “Yes” or “No” were used to determine the relationships among the indicators.

### 4.3. Developing a Structural Self-interaction Matrix (SSIM) and final reachability matrix (conical matrix)

An enabling relationship matrix was established as the structural self-interacted matrix (SSIM) in Table 6 below. The relationships between an indicator “a” and the other indicator “b” are defined as follows:

**Table 6:** Structural Self-interaction Matrix (SSIM).

	1	2	3	4	5	6	7	8	9	10	11	12	13	14	15	16	17	18	19	20
1		A	V	V	V	V	V	V	V	V	V	V	V	V	V	V	V	V	V	V
2			O	O	O	O	O	O	O	O	O	O	O	O	O	O	O	O	O	O
3				X	O	V	V	V	A	V	V	V	V	V	V	V	V	V	V	V
4					O	O	O	O	O	O	O	O	O	O	O	O	O	O	O	O
5						V	V	V	O	V	V	V	V	V	V	V	V	V	V	V
6							V	V	O	V	V	V	V	V	V	V	V	V	V	V
7								V	O	A	V	V	V	A	V	V	V	V	X	V
8									O	O	A	A	A	O	A	O	A	A	O	V
9										O	O	O	O	O	O	O	O	O	O	O
10											O	O	O	O	O	O	O	O	O	O
11												V	O	O	O	V	V	V	O	O
12													A	O	A	V	A	A	O	O
13														O	V	O	V	O	O	O
14															O	O	O	O	O	O
15																O	O	O	O	O
16																	O	O	O	V
17																		A	O	O
18																			O	O
19																				O
20																				

V → row indicator influences corresponding column indicator

A → row indicator is influenced by the corresponding column indicator

X → row and corresponding column indicators influence each other

O → row and corresponding column indicators have no relationship

The final reachability matrix (see Table 7) shows the driving and dependence powers for each indicator. The figures in each row, summed horizontally, represent the driving power, meaning how many other indicators (including itself) can influence or help accomplish. In contrast, the vertical total in each column shows the dependence power, which reflects how many other indicators (including itself) can contribute to achieving that particular indicator.

**Table 7:** Final Reachability Matrix.

	1	2	3	4	5	6	7	8	9	10	11	12	13	14	15	16	17	18	19	20	DP
1	1	0	1	1	1	1	1	1	1	1	1	1	1	1	1	1	1	1	1	1	**19**
2	1	1	1	1	1	1	1	1	1	1	1	1	1	1	1	1	1	1	1	1	**20**
3	0	0	1	1	0	1	1	1	0	1	1	1	1	1	1	1	1	1	1	1	**16**
4	0	0	1	1	0	1	1	1	0	1	1	1	1	1	1	1	1	1	1	1	**16**
5	0	0	0	0	1	1	1	1	0	1	1	1	1	1	1	1	1	1	1	1	**15**
6	0	0	0	0	0	1	1	1	0	1	1	1	1	1	1	1	1	1	1	1	**14**
7	0	0	0	0	0	0	1	1	0	0	1	1	1	0	1	1	1	1	1	1	**11**
8	0	0	0	0	0	0	0	1	0	0	0	0	0	0	0	0	0	0	0	1	**2**
9	0	0	1	1	0	1	1	1	1	1	1	1	1	1	1	1	1	1	1	1	**17**
10	0	0	0	0	0	0	1	1	0	1	1	1	1	0	1	1	1	1	1	1	**12**
11	0	0	0	0	0	0	0	1	0	0	1	1	0	0	0	1	1	1	0	1	**7**
12	0	0	0	0	0	0	0	1	0	0	0	1	0	0	0	1	0	0	0	1	**4**
13	0	0	0	0	0	0	0	1	0	0	0	1	1	0	1	1	1	0	0	1	**7**
14	0	0	0	0	0	0	1	1	0	0	1	1	1	1	1	1	1	1	1	1	**12**
15	0	0	0	0	0	0	0	1	0	0	0	1	0	0	1	1	0	0	0	1	**5**
16	0	0	0	0	0	0	0	0	0	0	0	0	0	0	0	1	0	0	0	1	**2**
17	0	0	0	0	0	0	0	1	0	0	0	1	0	0	0	1	1	0	0	1	**5**
18	0	0	0	0	0	0	0	1	0	0	0	1	0	0	0	1	1	1	0	1	**6**
19	0	0	0	0	0	0	1	1	0	0	1	1	1	0	1	1	1	1	1	1	**11**
20	0	0	0	0	0	0	0	0	0	0	0	0	0	0	0	0	0	0	0	1	**1**
**DEP**	**2**	**1**	**5**	**5**	**3**	**7**	**11**	**18**	**3**	**8**	**12**	**17**	**12**	**8**	**13**	**18**	**15**	**13**	**11**	**20**	

Note: DP: driving power; DEP: dependence power

### 4.4. The blended MOOC implementation model for English language learning

**Fig 3:**
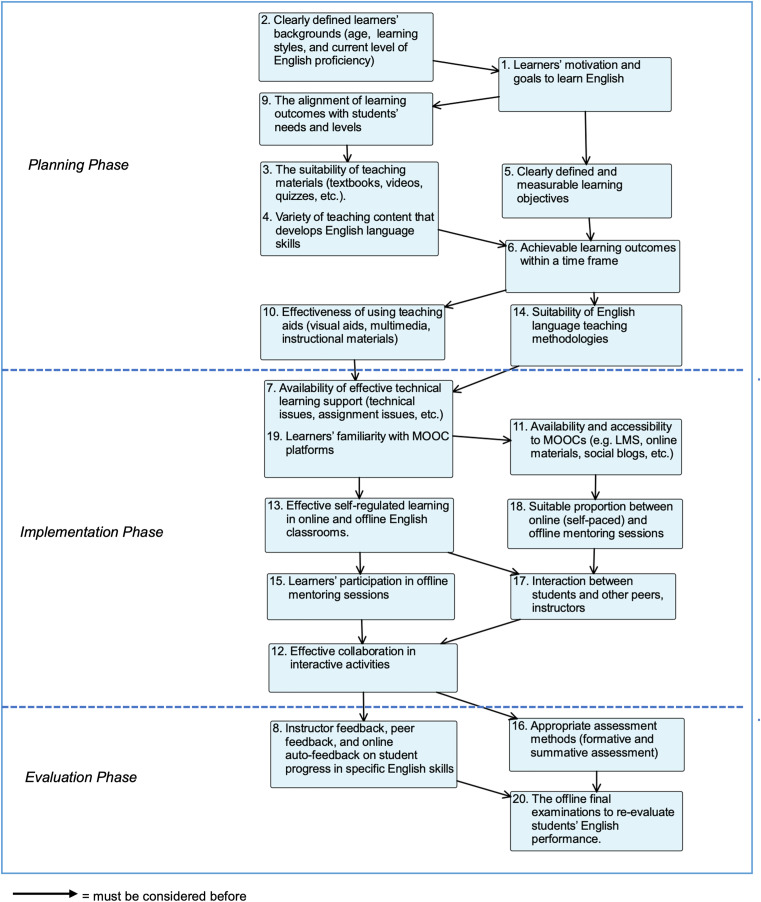
The proposed blended MOOC implementation model for English learning.

The model serves as a proposed guide for implementing MOOC-based blended learning in undergraduate English courses (see Fig 3). After the model was generated using the ISM software, the experts agreed to refine the model for improved suitability. The model was categorized into three phases: planning phase, implementation phase, and evaluation phase. The planning phase consists of all indicators and sub-indicators that help in understanding learners, setting goals, and designing learning content and objectives. The implementation phase includes all indicators and sub-indicators that facilitate course delivery, provide technical support, grant access to MOOCs, and encourage active participation and interaction. The evaluation phase encompasses all indicators and sub-indicators that assist in assessing student progress, delivering feedback, and conducting final assessments to ensure that learning outcomes are achieved. The arrows in the model indicate the movement from one indicator to another, following the hierarchical relationships, in a sequence of developing a blended MOOC implementation model for undergraduate English courses. Specifically, sub-indicator 1 must be considered before sub-indicators 5 and 9. In addition, the sub-indicators that share the same box mean that the sub-indicators could be considered in any sequence or simultaneously, as the sub-indicators complement one another (e.g., sub-indicators 3 and 4).

All the sub-indicators were partitioned into a reachability matrix. Partitioning the reachability matrix involves identifying, for each sub-indicator, its reachability set (elements it can influence) and antecedent set (elements that influence it). An element is assigned to a level if its reachability set is equal to the intersection of the reachability and antecedent sets. This process is repeated iteratively, removing assigned elements at each step, until all sub-indicators are organized into hierarchical levels (see Table 8). The sub-indicators were then categorized into hierarchical levels (See Table 9).

**Table 8:** Partitioning of reachability matrix.

Sub-indicators	Reachability Set	Antecedent Set	Intersection	Level
1	1,3,4,5,6,7,8,9,10,11,12,13,14,15,16,17,18,19,20	1,2	1	12
2	1,2,3,4,5,6,7,8,9,10,11,12,13,14,15,16,17,18,19,20	2	2	13
3	3,4,6,7,8,10,11,12,13,14,15,16,17,18,19,20	1,2,3,4,9	3, 4	10
4	3, 4, 6, 7, 8, 10, 11, 12, 13, 14, 15, 16, 17, 18, 19, 20	1, 2, 3, 4, 9	3, 4	10
5	5, 6, 7, 8, 10, 11, 12, 13, 14, 15, 16, 17, 18, 19, 20	1, 2, 5	5	10
6	6, 7, 8, 10, 11, 12, 13, 14, 15, 16, 17, 18, 19, 20	1, 2, 3, 4, 5, 6, 9	6	9
7	7, 8, 11, 12, 13, 15, 16, 17, 18, 19, 20	1, 2, 3, 4, 5, 6, 7, 8, 9, 10, 14, 19	7, 19	7
8	8, 20	1, 2, 3, 4, 5, 6, 7, 8, 9, 10, 11, 12, 13, 14, 15, 16, 17, 18, 19	8	2
9	3, 4, 6, 7, 8, 9, 10, 11, 12, 13, 14, 15, 16, 17, 18, 19, 20	1, 2, 9	9	11
10	7, 8, 10, 11, 12, 13, 15, 16, 17, 18, 19, 20	1, 2, 3, 4, 5, 6, 9, 10	10	8
11	8, 11, 12, 16, 17, 18, 20	1, 2, 3, 4, 5, 6, 7, 9, 10, 11, 14, 19	11	6
12	8, 12, 16, 20	1, 2, 3, 4, 5, 6, 7, 9, 10, 11, 12, 13, 14, 15, 17, 18, 19	12	3
13	8, 12, 13, 15, 16, 17, 20	1, 2, 3, 4, 5, 6, 7, 9, 10, 13, 14, 19	13	5
14	7, 8, 11, 12, 13, 14, 15, 16, 17, 18, 19, 20	1, 2, 3, 4, 5, 6, 9, 14	14	8
15	8, 12, 15, 16, 20	1, 2, 3, 4, 5, 6, 7, 9, 10, 13, 14, 15, 19	15	4
16	16,20	1, 2, 3, 4, 5, 6, 7, 9, 10, 11, 12, 13, 14, 15, 16, 17, 18, 19	16	2
17	8, 12, 16, 17, 20	1, 2, 3, 4, 5, 6, 7, 9, 10, 11, 13, 14, 17, 18, 19	17	4
18	8, 12, 16, 17, 18, 20	1, 2, 3, 4, 5, 6, 7, 9, 10, 11, 14, 18, 19	18	5
19	7, 8, 11, 12, 13, 15, 16, 17, 18, 19, 20	1, 2, 3, 4, 5, 6, 7, 9, 10, 14, 19	7, 19	7
20	20	1, 2, 3, 4, 5, 6, 7, 8, 9, 10, 11, 12, 13, 14, 15, 16, 17, 19, 19, 20	20	1

**Table 9:** Level partition of reachability matrix.

	Sub-indicators	Level
20	The offline final examinations to re-evaluate students’ English performance	1
8	Instructor and peer feedback + online auto-feedback on student progress in specific English skills	2
16	Appropriate assessment methods (formative and summative assessment)	2
12	Effective collaboration in interactive activities	3
15	Learners’ participation in offline mentoring sessions	4
17	Level of interaction between students and other peers, instructors	4
13	Effective self-regulated learning in online and offline English classrooms	5
18	Suitable proportion between online (self-paced) activities and offline mentoring activities	5
11	Availability and accessibility to MOOCs (e.g., LMS, online materials, social blogs, etc.)	6
7	Availability of effective technical learning support (technical issues, assignment issues, etc.)	7
19	Learners’ familiarity with MOOC platforms	7
10	Effectiveness of using teaching aids (visual aids, multimedia, instructional materials)	8
14	Suitability of English language teaching methodologies	8
6	Achievable learning outcomes within a time frame	9
3	The suitability of teaching materials (textbooks, videos, quizzes, etc.)	10
4	Variety of teaching content that develops English language skills	10
5	Clearly defined and measurable learning objectives	10
9	The alignment of learning outcomes with students’ needs and levels	11
1	Learners’ motivation and goals to learn	12
2	English Clearly defined learners’ backgrounds (age, learning styles, and current level of English proficiency)	13

**Fig 4:**
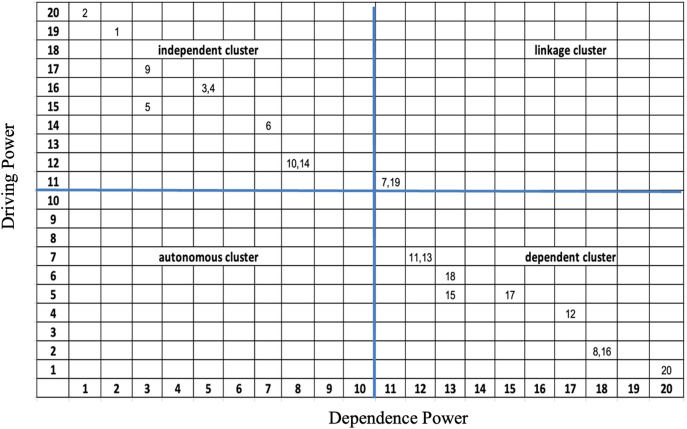
Matrice d’Impacts Croises Multipl ication Applique a Classement (MICMAC) Analysis for sub-indicators.

The MICMAC (Cross-Impact Matrix Multiplication Applied to Classification) analysis for the blended MOOC implementation model for English language learning, using the ISM (Interpretive Structural Modelling) method, reveals how different indicators and sub-indicators relate to each other in terms of influence and dependence. As in Fig 4, the results indicate that most key sub-indicators (1, 2, 3, 4, 5, 6, and 9) fall within the independent sub-indicators area, which suggests they have a strong influence on the system but depend less on other sub-indicators. These should be given priority when designing the model, as improving them would have a significant overall impact. Besides, two sub-indicators (7 and 19) fall into the linkage cluster, meaning they are both influential and dependent since they are sensitive and should be carefully considered, because any change can affect the system. Sub-indicators 11, 12, 13, 15, 16, 17, 18, and 20 are dependent sub-indicators; they rely on other factors and mainly show results or outcomes of the model. Finally, there are no autonomous sub-indicators, showing that most sub-indicators are connected in some way and none are isolated. This structure highlights that focusing on strong, independent drivers could help build a hierarchical blended MOOC implementation model for English learning. The results reveal that learner analysis and institutional support are the key driving factors with high driving power and low dependence. These elements form the foundational layer of the model. In contrast, learner outcomes and engagement-related factors exhibit high dependence power, indicating that they are largely influenced by other elements in the system.

## 5. Discussion

The development of a blended MOOC implementation model for undergraduate English learning was based on six main indicators: analysis of learners, objectives, course materials and technologies, teaching methodologies, learners’ participation, and feedback and assessment. As in Fig 3, the model was classified into three phases: planning phase, implementation phase and evaluation phase.

The planning phase underscores learner analysis as the key driver of the model. “Clearly defined learners’ backgrounds” (sub-indicator 2) and “learners’ motivation and goals to learn English” (sub-indicator 1) were ranked highest in the independent cluster in the MICMAC analysis (Fig 4), indicating a high level of influence within the system. These findings suggest that effective blended MOOC design must begin with a clear understanding of learner profiles, proficiency levels, learning preferences, and motivational factors. In EFL contexts, where learning progress depends heavily on sustained practice and interaction, inadequate learner analysis is often associated with misalignment between course design and learner needs. Motivation is also perceived to influence engagement and persistence, which are critical in MOOC-based blended environments where self-regulation is required. The prominence of these learner-related indicators, therefore, highlights the central role of pre-instructional analysis in ensuring effective and sustainable blended MOOC implementation. These findings are consistent with prior studies that emphasize the importance of learner analysis in MOOC design, where understanding learner characteristics significantly improves alignment between instructional design and learner needs [55,56,58].

The indicator “objectives” also emerged as influential in the planning phase, particularly “clearly defined and measurable learning objectives” (sub-indicator 5). Teaching materials and content (sub-indicators 3 and 4) were also highly ranked, emphasizing the need for high-quality, accessible, and linguistically appropriate resources. Clear learning outcomes and suitable materials should be established before selecting teaching methodologies to ensure instructional coherence and alignment with learner needs [61,62,64]. In EFL MOOCs, well-designed videos, quizzes, and reading materials support the development of both receptive and productive skills and accommodate learners with different proficiency levels [10,23]. This sequencing highlights that instructional methods should be selected only after goals and materials are clearly defined, preventing unfocused or ineffective teaching practices in technology-mediated learning environments. This finding aligns with previous research highlighting that clearly defined objectives and well-structured materials are critical for maintaining instructional coherence in blended and online learning environments [61,62].

The second key finding highlights the importance of technology in the implementation phase. “Technical learning support” and “learners’ familiarity with MOOC platforms” (sub-indicators 7 and 19) were identified as linkage variables in the MICMAC analysis, indicating that technological access and platform familiarity play a central role in maintaining the stability of the blended MOOC model [65]. These indicators are both influential and highly dependent, suggesting that changes in surrounding instructional or support conditions may be associated with changes in the overall performance of the model. Without adequate infrastructure and platform familiarity, learners may experience technical barriers that interrupt learning continuity and reduce engagement. This result supports earlier findings that technological readiness and platform usability are key determinants of learner engagement and persistence in MOOCs [65].

In addition, “availability and accessibility of MOOCs” (sub-indicator 11), together with built-in support mechanisms, are considered important for reducing cognitive overload and sustaining learner engagement [48,69]. Peer and instructor interaction (sub-indicator 17) and offline mentoring sessions (sub-indicator 15) further promote active participation, social presence, and collaborative learning in EFL blended MOOCs. These design features reflect a shift away from passive video-based learning typical of MOOCs toward more communicative and socially grounded learning environments [19,52]. Meaningful interaction fosters a sense of community and supports deeper learning, which are commonly linked to motivation and retention [67]. This finding highlights that technological elements in blended MOOCs should be designed not only for content delivery but also to facilitate interaction and guided support.

The third major finding relates to the evaluation phase, where appropriate assessment methods (sub-indicator 16) and instructor and peer feedback (sub-indicator 8) were rated more important than traditional offline final examinations (sub-indicator 20), which had the lowest driving and independent power in the MICMAC analysis. This result suggests the use of formative and continuous assessment with timely feedback as more suitable approaches for EFL blended MOOCs, as they reduce learner isolation, support self-regulation, and better reflect gradual language development than summative final examinations [71,73,77]. In language learning, progress is often incremental and skill-specific; therefore, relying solely on final examinations may not fully capture learners’ continuous improvement in listening, speaking, reading, and writing skills. The lower ranking of offline final examinations further indicates a shift toward learner-centred evaluation practices that emphasize learning processes rather than single high-stakes assessments. This result reinforces previous studies suggesting that formative assessment and continuous feedback are more effective than summative approaches in supporting language development in EFL contexts [71,73].

More interestingly, the proposed model would be beneficial to a real educational EFL setting through three phases. In the planning phase, instructors first review learner profiles using placement tests and short surveys to identify students’ English levels, learning preferences, and motivation. Based on this information, clear learning objectives are set, such as improving academic writing coherence. Suitable MOOCs on writing and grammar are then selected as the main online learning materials. In the implementation phase, students watch MOOC videos and complete online quizzes before attending weekly face-to-face mentoring sessions. Classroom time is used for guided writing practice, peer feedback, and teacher support. Technical assistance is provided through the learning management system, and student participation is encouraged through group activities and regular progress checks. In the evaluation phase, student learning is monitored using formative quizzes, writing portfolios, and peer-feedback rubrics rather than relying only on a final examination. Continuous feedback enables instructors to adjust teaching activities and provide additional support to students who show low engagement or slow progress. This guidance shows how the proposed model can guide practical teaching decisions in real EFL classroom settings. Compared to previous studies, this research contributes by offering a structured ISM-based model that not only identifies key indicators but also explains their hierarchical relationships and influence within the system.

## 6. Conclusion

This study presents a structured and validated model for implementing blended MOOCs in undergraduate English language learning. Developed through the Interpretive Structural Modelling (ISM), the model is organized into three phases: planning, implementation, and evaluation. In the planning phase, understanding learners’ backgrounds, needs, and motivations is emphasized as the foundation for designing effective instruction. Clear learning objectives and suitable, engaging course materials are identified as essential components for aligning pedagogy with learner expectations. The implementation phase highlights the importance of accessible technology, appropriate teaching methodologies, and opportunities for active learner participation, both online and face-to-face. Technical support and familiarity with MOOC platforms are also critical for sustaining engagement. In the evaluation phase, the model prioritizes formative assessment and continuous feedback over traditional final examinations, promoting a more supportive and responsive learning environment. This learner-centered approach ensures that instruction is flexible, inclusive, and aligned with contemporary practices in digital education. The proposed model addresses key challenges in EFL instruction by bridging the gap between traditional teaching and online learning through the mode of blended MOOCs. It offers a practical guideline for diverse stakeholders, such as educators, curriculum designers, and institutional leaders seeking to enhance English language learning.

## 7. Implications, limitations, and recommendations

The proposed blended MOOC implementation model offers practical implications grounded in the hierarchical structure identified through ISM analysis. Elements with high driving power should be prioritized, as they form the foundation for subsequent stages. At the foundational level, learner analysis, learning objectives, and content and technology selection were identified as primary drivers. Therefore, educators and administrators should prioritize understanding learner characteristics and aligning course objectives before investing in advanced instructional strategies or engagement tools. At the implementation level, instructional strategies and learner participation depend on these foundational elements. This indicates that efforts to enhance engagement will only be effective when clear objectives and appropriate technological support are already in place. At the evaluation level, assessment and feedback were found to be highly dependent elements. Thus, timely feedback and formative assessment should be strengthened after the successful implementation of planning and instructional processes. By following this hierarchy, institutions can address common challenges in bMOOCs, such as low student motivation and high dropout rates. For example, prioritizing learner needs and appropriate instructional design can reduce learner frustration and disengagement, thereby improving retention and overall learning outcomes.

While the proposed blended MOOC implementation model offers a structured approach to English instruction in higher education, several limitations should be acknowledged. Firstly, the model was developed based on expert input and literature analysis within the specific context of Vietnamese higher education. This context-specific design may limit the generalizability of the findings to other educational systems with different technological infrastructures or pedagogical traditions. Local institutional conditions such as large class sizes, exam-oriented curricula, and policy-driven English requirements may influence the relevance and priority of some indicators. Therefore, the direct transfer of the model to other national or disciplinary contexts may require contextual adaptation. The second limitation is that this study focused on expert validation rather than large-scale classroom implementation. Although the indicators were validated through expert consensus, empirical testing is still needed to examine the model’s suitability in real EFL teaching contexts and to evaluate its effects on learner engagement and learning outcomes over time. In particular, the absence of longitudinal classroom data makes it difficult to assess the long-term impact of the model on learner engagement and language proficiency development. Finally, the rapid evolution of educational technology, particularly artificial intelligence–based learning tools, may influence the long-term applicability of some indicators and sub-indicators. Future research should pilot the model in diverse institutional settings to assess its practical effectiveness and scalability. Longitudinal studies involving both educators and learners could provide deeper insights into how the model supports language acquisition and sustained engagement. Furthermore, future studies may investigate the integration of AI-driven learning analytics and personalized feedback mechanisms to improve learner support and progression tracking in blended MOOC environments.
